# Phytochemical Analysis, Cytotoxic, Antioxidant, and Antibacterial Activities of Lichens

**DOI:** 10.1155/2020/8104538

**Published:** 2020-12-02

**Authors:** Noura Aoussar, Fatima Ezzahra Laasri, Mohammed Bourhia, Nedeljko Manoljovic, Rajaa Ait Mhand, Naima Rhallabi, Riaz Ullah, Abdelaaty A. Shahat, Omar M. Noman, Fahd A Nasr, Omer M. Almarfadi, Mohammed El Mzibri, Perica Vasiljević, Laila Benbacer, Fouad Mellouki

**Affiliations:** ^1^RU Microbiology, Hygiene and Bioactive Molecules, LVMQB/EB, University Hassan II of Casablanca, Faculty of Sciences and Techniques of Mohammedia, Casablanca 20650, Morocco; ^2^Unit of Biology and Medical Research, National Center for Energy, Nuclear Science and Technology, Rabat, Morocco; ^3^Laboratory of Chemistry-Biochemistry, Environment, Nutrition, and Health, Faculty of Medicine and Pharmacy, University of Hassan II, B.P, Casablanca 5696, Morocco; ^4^Department of Pharmacy, Faculty of Medical Sciences, University of Kragujevac, Kragujevac 34000, Serbia; ^5^Department of Pharmacognosy (MAPPRC), College of Pharmacy, King Saud University, Riyadh, Saudi Arabia; ^6^Chemistry of Medicinal Plants Department, National Research Centre, P. No. 33 El Bohouth St. (former El Tahrirst.), Giza 12622, Dokki, Egypt; ^7^Department of Biology and Ecology, Faculty of Sciences and Mathematics, University of Nis, Nis 18000, Serbia

## Abstract

**Background:**

Lichens present a complex symbiotic relationship between a filamentous fungus, photoautotrophic partner (algae or cyanobacteria), and bacterial community. *The Objective of the Study*. This study aimed at investigating the chemical composition and cytotoxic, antioxidant, and antimicrobial activities of acetone extracts of Moroccan *Evernia prunastri (E. prunastri), Ramalina farinacea* (*R. farinacea*), and *Pseudevernia furfuracea (P. furfuracea). Materials and Methods*. The phytochemical analysis was carried out by HPLC-UV. The cytotoxic effect was assessed on human prostate cancer (22RV1), human colon carcinoma (HT-29), human hepatocellular carcinoma (Hep-G2), and Hamster ovarian cancer (CHO) cells lines by WST1 assay. The antioxidant power was assessed by DPPH and FRAP assays. The antibacterial effect was obtained using the broth microdilution method.

**Results:**

The findings of phytochemical analysis showed that the lichens studied possess interesting bioactive molecules such as physodalic acid, evernic acid, and usnic acid, as well as protocetraric acid. According to the American National Cancer Institute guidelines, the WST-1 test showed that all crude extracts did not show significant cytotoxic effects against all concerous cell lines, and IC_50_ values ranged from 42.30 to 140.24 *µ*g/mL. Regarding the antioxidant activity, *P. furfuracea* extract showed the highest free-radical-scavenging ability (IC_50_ = 498.40 *µ*g/mL). The most potent antibacterial extract was recorded for *P. furfuracea* extract with a minimum inhibitory concentration (MIC) ranging from 0.039 to 0.31 mg/mL.

**Conclusion:**

In this research work, we report that the studied lichen extracts exhibit an important biological effect, supporting that lichens represent a hopeful source of original natural products for the research of new bioactive molecules having a pharmaceutical interest.

## 1. Introduction

Lichens are naturally arising from an alliance between fungus and algae [1, 2]. Moreover, bacteria can also colonize lichens to form a third partner [3, 4]. There are about 18,500 species of lichens worldwide that can survive in various extreme environmental conditions due to their exceptional resistance capacity that makes them pioneer species. The intrinsic resistance of lichen is mainly due to the production of a wide range of compounds derived typically from secondary metabolites of fungal components which build up in the cortex or the medullary layer [5, 6]. Approximately 1050 chemical substances are identified in lichens including depsides, depsidones, and dibenzofurans [2]. Lichens are known for their several medicinal virtues, and their metabolites have been described for their multiple biological properties [5, 7, 8].

Currently, in cancer treatment, several anticancer drugs have been used with important side effects due to their close therapeutic margin and high toxicity. Moreover, the risks for infection are increased due to the small number of white blood cells (neutropenia) arising from the chemotherapy toxic effect on the bone marrow [9]. Due to its immunocompromised status, antimicrobial therapy is often undertaken in hospitalized cancer patients. However, the significant increase in use of antibiotics is associated with the appearance of multidrug-resistant pathogens such as *Staphylococcus aureus.* This bacterium is the main nosocomial pathogen agent worldwide and the most worrisome, particularly *S. aureus* resisting the methicillin (MRSA), as well as it is easily capable to develop in biofilms in hospitalized patients [10].

Furthermore, oxidative stress induced by the excessive production of free radicals is associated with different chronic diseases and also to almost many cancers; namely, in tumor progression [11], the search for natural antioxidant compounds is of great interest to preserve the physiological performances of the body.

To overcome these issues, the researchers are ardently seeking alternative bioactive molecules (antimicrobial, antioxidant, and anticancer), with high efficacy and fewer secondary effects. Lichen secondary metabolites have been documented widely for their effectiveness against different tumor cells and also for their bacterial resistance potential. As far as we can tell, a few studies have evaluated the anticancer activity of *Evernia prunastri, Pseudevernia furfuracea*, and *Ramalina farinacea.* However, the Moroccan lichens have not yet been studied in terms of pharmacological effects.

The present research study aimed to investigate *in vitro* antioxidant potency and antimicrobial, as well as cytotoxic, effects of organic extracts from *E. prunastri, P. furfuracea*, and *R. farinacea* growing in Moroccan soil.

## 2. Materials and Methods

### 2.1. Lichen Material

Thallus samples of *R. farinacea* (L.) Ach., *E. prunastri* (L.) Ach., and *P. furfuracea* (L.) Zopf. were collected from Khenifra, Morocco. The collected lichens were identified based on morphological characteristics determined by macroscopic and microscopic studies, as well as on the basis of colorful reactions by chemical reagents [12]. Voucher specimens of collected species (*P. furfuracea* # 2501, *E. prunastri* # 2502, and *R. farinacea* # 2503) have been put at the Herbarium of Moroccan Scientific Institute.

### 2.2. Preparation of Lichen Extracts

Thalli of three lichen species were dried and ground into a fine powder. The powder was extracted by maceration (24 h) using acetone at ambient temperature [13]. Extracts of species were filtered then concentrated at 40°C under reduced pressure. The extraction yield obtained 4.52%, 1.32%, and 4.32% for *P. furfuracea, R. farinacea*, and *E. prunastri* extracts, respectively. The extracts obtained were kept at −20°C until further analysis.

### 2.3. HPL Analysis

HPLC-UV analysis was performed according to the method adopted by Huneck and Yoshimura [14]. Extracts were solubilized in acetone (500 *μ*L), and the analysis was performed using HPLC (Agilent Technologies, 1200 Series). An injection volume of 10 *μ*L of the extract was analyzed using a mobile phase consisting of methanol-water-phosphoric acid in the presence of a detector of UV spectrophotometer (254 nm). Deionized water was purified using a purification system (Milli-Q.). HPLC-grade methanol was purchased from Merck (Darmstadt, Germany). The identification of polyphenolic compounds contained in extracts was carried out by comparing retention times (*t*_R_) and absorption spectra (200–400 nm) with those of the authentic substances isolated early from other lichen species. Previous studies have shown that the three tested lichens contain certain phenolic acids (evernic acid, fumarprotocetraric acid, atranorin, usnic acid, physodalic acid, chloroatranorin, and protocetraric acid), and that is why we chose them to be used as reference compounds. The standards used in this study were acquired from the following sources: evernic acid and atranorin are isolated from the *Evernia prunastri* [15], fumarprotocetraric acid was purified from *C. rangiferina* and usnic acid from *Cladonia foliacea* [16], physodalic acid and chloroatranorin from *Hypogymnia physodes* [17], and protocetraric acid from *Toninia candida* [18].

### 2.4. *In Vitro* Cytotoxic Activity

#### 2.4.1. Cell Lines and Culture

Human prostate cancer (22RV1) cells were kindly provided by Dr. Belharazem, Institute of Pathology, Medical Faculty of Mannheim University, Heidelberg. Human colon carcinoma (HT-29), human hepatocellular carcinoma (Hep-G2), and hamster ovarian cancer (CHO) cell lines were kindly given by Dr. L'Houcine, OUAFIK, APHM, North Hospital, Transfer Laboratory, Marseille 13015, France. These cell lines were maintained and cultured as a monolayer in a DMEM medium with the following components: inactivated fetal calf serum with 10%, glutamine with 1%, and antibiotics with 1%, except for CHO cell lines that were maintained in McCoy's 5 A medium. The cells were grown at 37°C in a wet atmosphere with air (95%) and CO_2_ (5%).

#### 2.4.2. Cell Viability Assay

The cytotoxic effect of the acetone extracts of *P. furfuracea, R. farinacea*, and *E. prunastri* against cancer cell lines was estimated using the WST1 test [19]. All cell lines were regularly seeded in 96-well microplates. After cell adhesion (24 h), the five different extract concentrations, 200 *μ*g/mL, 100 *μ*g/mL, 50 *μ*g/mL, 25 *μ*g/mL, and 12.5 *μ*g/mL, were added in duplicate to the wells and reincubated. After incubation at 37°C for 72 h, 100 *μ*L of the medium was replaced with 10 *μ* L of WST1 and incubated again for further time. Mitomycin was used as a drug reference, and results were presented as the percentage of cell viability, which was determined via the following equation:

(1) $$\begin{array}{c} \text{Cell viability}\left(\text{\%}\right)=\left(\frac{{\text{A}}_{\text{sample}}}{{\text{A}}_{\text{Control}}}\right)\times 100\text{.} \end{array}$$

A_sample_ and A_Control_ with and without extract, respectively, were read for the assessment of absorbance. The test was evaluated in duplicate.

### 2.5. Antioxidant Activity

#### 2.5.1. FRAP Assay

The ferric-reducing powers of lichen extracts were evaluated according to the method described in the early literature [20]. In brief, 1 mL of extract (50–1000 *µ*g/mL) was mixed with 2.5 mL of phosphate buffer and then added to 2.5 ml of the solution of potassium ferricyanide (1%). Afterward, the mixture was incubated for 30 min at 50°C and then centrifuged at 3000 rpm. 2.5 mL of the supernatants were added to 2.5 mL of distilled water and mixed with 0.1% FeCl_3_. Finally, the absorbance of the resulting solutions was recorded at 700 nm. In this assay, trolox and ascorbic acid were used as standards. Increasing the absorption of the sample is an indication of increasing reducing power. All experiments were executed in triplicate.

#### 2.5.2. DPPH Assay

The measurement of the antiradical effect of extracts from the studied lichen species was carried out by the DPPH test as described by Kosanić et al. [21]. Briefly, 1 mL of extract (50–1000 *µ*g/mL) was mixed with 2 mL of DPPH aliquot (0.12 mM). The reaction mixture was incubated for 25 min in the dark at ambient temperature. The absorbance of the mixture was recorded at 517 nm. The percentage of inhibition of the DPPH radical was performed using the equation given below.

Scavenging of DPPH (%) = 100 × [(Absorbance of blank − Absorbance of the sample)/Absorbance of blank].

IC_50_ values were obtained from the percentage inhibition vs. concentration plot, using Regtox software, and expressed in *μ*g/mL. All measurements were conducted in triplicate.

#### 2.5.3. Determination of Phenols

Total phenolic content (TPC) in extracts was meticulously assessed using the Folin–Ciocalteu method [22], with some modifications. Briefly, 100 *µ*L of extracts (1 mg/mL) was diluted up to 4.6 mL and then added to100 *µ*L of the reagent of Folin–Ciocalteu. Afterward, the mixture was left for 3 min and then added to Na_2_CO_3_ (300 *µ*L, 2%). After incubation for 90 min at 25°C, the absorbance was read at 760 nm. Results were expressed as *µ*g GAE/mg dry extract.

#### 2.5.4. Determination of Total Flavonoid Content

Total flavonoid content (TFC) in extracts was evaluated using protocols as previously described [23]. An aliquot of 500 microliters of each lichen extract (1 mg/mL) was added to 75 *µ*L of sodium nitrite solution (5%) mixed with 150 *µ*L of aluminum chloride (10%), after 5 min at ambient temperature, 500 *µ*L of NaOH reagent (1 M) was added, and then, the absorbance was recorded at 510 nm. TFC was presented as catechin equivalent (CE) (*µ*g CE/mg of dry extract).

### 2.6. Antimicrobial Activity

#### 2.6.1. Bacterial Strains

The antibacterial activity of lichen extracts was assessed against 11 bacterial strains including Gram-positive bacteria: *S. aureus (*ATCC 25923), five clinical Methicillin-Resistant *S. aureus* (MRSA) isolates from burn wounds of patients at IbnRochd University Hospital of Casablanca (Morocco), *Listeria innocua* (CECT 4030)*, B. subtilis* (DSM 6633), and Gram-negative bacteria, namely, *Escherichia coli* (ATCC 25922), *P. aeruginosa* (CECT 118), *P. mirabilis.*

The *S. aureus* clinical isolates were identified as multidrug resistant by testing their antibiotic susceptibility according to the EUCAST 2016 guidelines [24], as described by Achmit et al. [25].

#### 2.6.2. Determination of MIC and MBC

The MICs (Minimum Inhibitory Concentrations) were determined according to data by Satyajit et al. with some modifications [26]. Wells of the plate were filled with both culture medium and extracts (v/v: 100/100 *µ*L) at concentrations ranging from 5 to 0.002 mg/mL; to each well, bacterial inoculum at 5 × 10^6^ CFU/mL was added followed by resazurin solution (0.015%) as a marker of microbial growth. The plates were incubated again for 24 h at 37°C. The lowest effective concentration was considered as a minimal inhibitory concentration (MIC) [27]. Experiments were realized in duplicate.

Regarding the MBC (Minimum Bactericidal Concentration), 10 *µ*L from purple wells of the MICs test were subcultured on nutrient agar in Petri plates. MBC was considered as the lowest effective concentration with no bacterial growth after reincubation. Moreover, for each extract, the ratio CMB/MIC was calculated to assess its antibacterial ability, the extract has a bactericidal effect when CMB/MIC = 1–2 and a bacteriostatic effect when CMB/CMI = 4–16 [28].

### 2.7. Statistical Analysis

Data were reported as mean ± (SD). One-way ANOVA and post hoc *t*-tests were used for statistical analysis. The correlation coefficient was defined by the Pearson test using SPSS-22. The differences were accepted as significant at *p* < 0.05.

## 3. Results

### 3.1. HPLC Analysis

**Figure 1:**
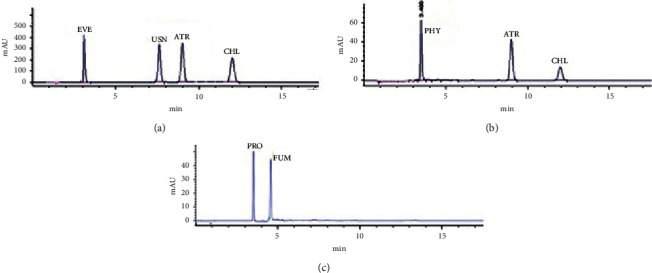
HPLC chromatograms of the standards used at 254 nm. EVE—evernic acid, USN—usnic acid, ATR—atranorin, CHL—chloratranorin, PHY—physodalic acid, PRO—protocetraric acid, and FUM—fumaprotocetraric acid.

**Figure 2:**
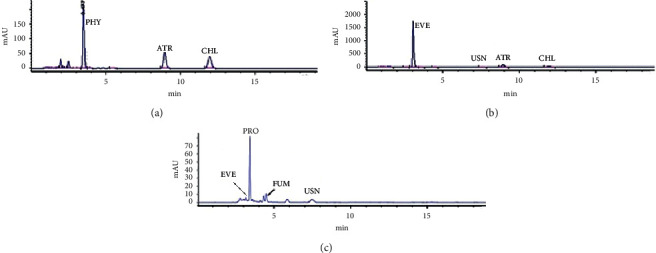
HPLC chromatograms of extracts of *Pseudevernia furfuracea* (a), *Evernia prunastri* (b), and *Ramalina farinacea* (c) at 254 nm.

**Figure 3:**
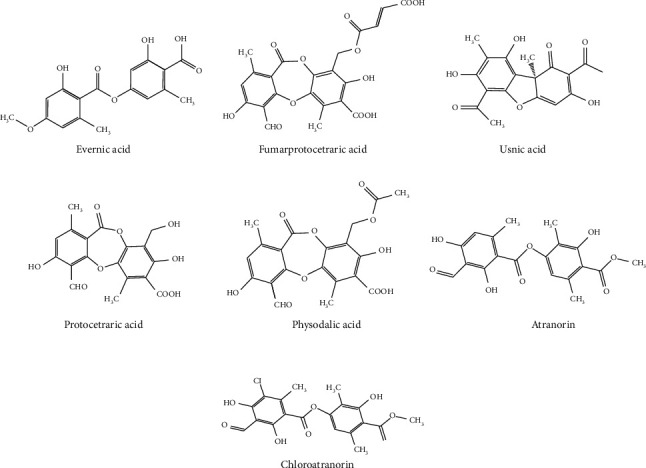
The chemical structures of the identified compounds.

The HPLC-UV analysis of extracts of *R. farinacea, E. prunastri*, and *P. furfuracea* was used to identify their main phenolic acids by matching their retention times (*t*_R_) and absorbance maxima (nm) UV spectrum with the reference compounds. The chromatograms of eleven standards and extract samples are given in Figures 1 and 2. The structures of the identified molecules are shown in Figure 3. The obtained data confirmed that the main compounds in extracts of *P. furfuracea* were physodalic acid (PHY), atranorin (ATR), and chloratranorin (CHL). PHY was the most abundant substance. Evernic acid (EVE), usnic acid (USN), atranorin (ATR), and chloratranorin (CHL) were identified, with EVE being the most abundant compound in *E. prunastri*. Protocetraric acid (PRO), fumarprotocetraric acid (FUM), EVE, USN, and ATR were identified, with PRO being the predominant phenolic compound in *R. farinacea* (Figure 2).

### 3.2. Cytotoxic Activity

**Figure 4:**
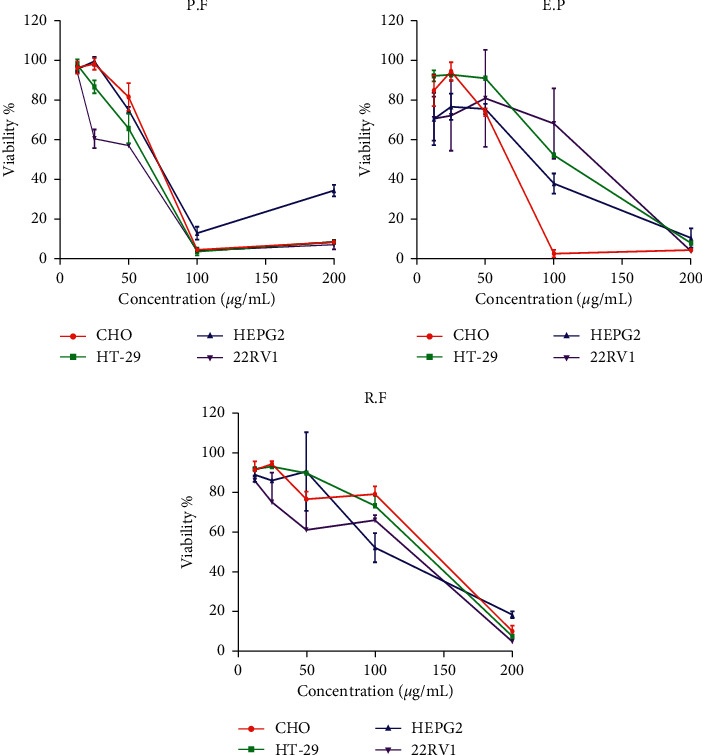
: Percentage of cell viability of CHO, HT-29, HEP-G2, and 22RV1 cell lines treated with varying concentrations of extracts of *P. furfuracea* (P.F), R*. farinacea* (R.F), and *E. prunastri* (E.P) for 72 h.

**Figure 5:**
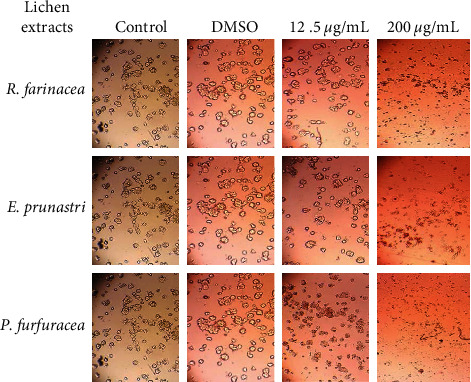
Morphological aspects of HT-29 cells before (Control) and after 72 h treatment with acetone extract of *R. farinacea*, *E. prunastri*, and *P. furfuracea* with 12.5 *µ*g/mL and 200 *µ*g/mL concentrations and with DMSO at 200 *µ*g/mL.

The cytotoxic effect of *R. farinacea, E. prunastri*, and *P. furfuracea* extracts against different cell lines was assessed using the WST1. The results revealed that the extracts demonstrated a relatively low cytotoxic effect against all cells in a dose-dependent manner (Figure 4). Loss of cell viability was revealed by the morphological and aggregation changes depending on the concentration of extracts as shown, for example, by the extracts of *R. farinacea, E. prunastri,* and *P. furfuracea* against HT-29 cell lines (Figure 5). As shown in Figure 5, the number of dead cells positively correlates with the concentration of the extracts. At high concentrations of the extracts, cells started to get a more enlarged shape and a formation of blebs in the cell's membranes. We also noticed the appearance of apoptotic bodies, large vacuoles in the cell cytoplasm, and rounded shape of the cells that start to detach from the surface and float in the medium indicating cell death. The IC_50_ values of organic extracts from lichens ranged from 42.30 to 140.24 *µ*g/mL (Table 1) with no significant difference between the sensitivity of cancer cells treated by *E. prunastri* and by *R. farinacea* (*p* > 0.05), and for *P. furfuracea*, we found a significant difference between 22RV1 cells and the other cell lines (*p* < 0.05). Furthermore, a significant difference was observed between *P. furfuracea* and *R. farinacea* in the inhibition of all tested cell lines and between *P. furfuracea* and *E. prunastri* for HT-29 and 22RV1 cells (*p* < 0.05). Among extracts studied, *P. furfuracea* extract was found to induce the largest effect towards all cancer cell lines tested, especially against 22RV1 (human prostate cancer) cells. As can be seen in Table 1, the cytotoxic effect of extracts studied was lower compared to that of mitomycin (positive control).

### 3.3. Antioxidant Activity

The total phenolic contents (TPC) of *E. prunastri, P. furfuracea*, and *R. farinacea* extracts were calculated using the gallic acid curve (*R*^2^ = 0.99). As shown in Table 2, the TPC of the three lichen extracts ranged from 167.67 to 328.67 *μ*g GAE/mg of dry extract. *P. furfuracea* extract showed the highest TPC (328.67 *μ*g GAE/mg of crude extract). We found a significant difference between *P. furfuracea* and *E.prunastri*-*R.farinacea* (*p* < 0.05) but not between *R. farinacea* and *E. prunastri* (*p* > 0.05). TFC of these extracts was calculated from the catechin calibration curve (*R*^2^ = 0.97). The TFC of tested extracts ranged from 12.23 to 17.63 *μ*g CE/mg of the dry extract with a significant difference between *R. farinacea* and *P. furfuracea*-*E. prunastri* (*p* < 0.05), but no significant difference between *P. furfuracea* and *E. prunastri* was reported (*p* > 0.05). The highest total flavonoid content was registered for the extract of *R. farinacea* (Table 2).

**Figure 6:**
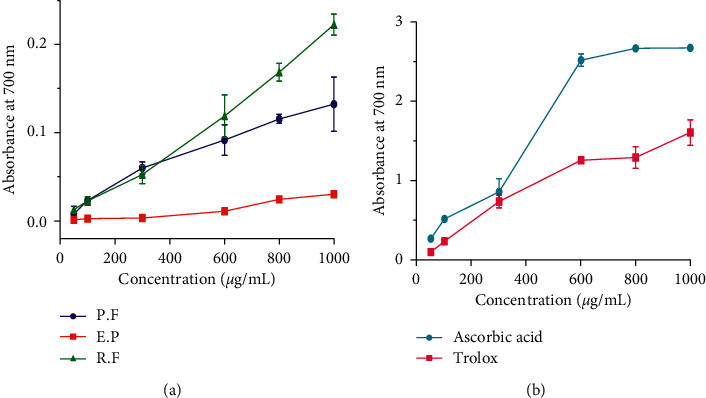
Reducing power of the lichens *R. farinacea* (R.F), *E. prunastri* (E.P), and *P. furfuracea* (P.F) extracts (a) and ascorbic acid and Trolox (b).

The ferric reducing power of the studied crude extracts was reported in a dose-dependent manner. As shown in Figure 6, the highest activity was obtained for *R. farinacea* extract with absorbance increased from 0.01 to 0.22. However, no significant difference between the extract of *R. farinacea* and *P. furfuracea* was observed. This activity remains lower compared to the positive controls (ascorbic acid and Trolox) (Figure 6).

**Figure 7:**
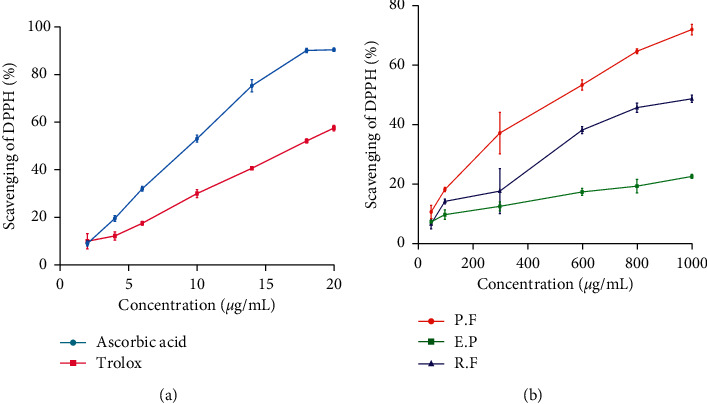
Scavenging effect of standards, ascorbic acid, and Trolox (a) and *P. furfuracea* (P.F), *E. prunastri* (E.P), and *R. farinacea* (R.F) (b).

The DPPH test of lichen extracts was performed, and the obtained results are reported in Figure 7. All lichen extracts exhibited strong scavenging ability which varied from 6.63% to 72.12% for concentrations ranged from 50 to 1000 *μ*g/mL, with a significant correlation with TPC (*r* = 0.69). Among the tested extracts, *P. furfuracea* extract showed the best scavenging effect (IC_50_ = 498.40 *µ*g/mL), which was significantly different than *R. farinacea* and *E. prunastri* (*p* < 0.05). The results also showed that the standards (ascorbic acid and Trolox) demonstrated stronger DPPH radical-scavenging activity than the tested extracts (Table 3).

### 3.4. Antibacterial Activity

The antibacterial effect of *R. farinacea*, *E. prunastri*, and *P. furfuracea* extracts was evaluated by the microdilution method with resazurin vs. eleven bacterial strains including 5 clinical isolates of methicillin-resistant *S. aureus*. The MIC and the MBC of extracts were determined, and the results are presented in Table 4. These findings revealed that all extracts exhibited a higher antibacterial effect vs. Gram-positive bacteria. However, no effect was recorded for Gram-negative bacteria. *P. furfuracea* exhibited an antibacterial effect with MIC values of 0.039–0.15 mg/mL and MBC 0.625 mg/mL for all strains. The extract from *E. prunastri* presented a MIC ranged from 0.039 to 0.15 mg/mL and MBC from 0.625 to 2.5 mg/mL; also, the extract of *R. farinacea* possessed MIC in the range of 0.078–0.625 mg/mL, while its MBC was at 0.625–1.25 mg/mL.

The lower MIC value was demonstrated for *P. furfuracea* and *E. prunastri* in the SARM strain N°1 (0.039 mg/mL), and the higher MBC value was found for *E. prunastri* in *Listeria innocua* (2.5 mg/mL). Overall, the MIC values obtained for the acetone extract of *P. furfuracea* were lower than those obtained with extracts of *E.prunastri* and *R.farinacea*.

From the obtained ratio, MBC/MCI, it can be noticed that the extract from *P. furfuracea* showed a bactericidal effect against *Listeria innocua* and for *R. farinacea* against strains of MRSA N°2, 3, 4, and 5. For the rest of the strains, a bacteriostatic effect was recorded.

## 4. Discussion

In the present research work, we examined chemical profiles and the *in vitro* cytotoxic, antioxidant, and antimicrobial activities of organic extract from Moroccan lichens, *R. farinacea, E. prunastri* and *P. furfuracea*. The studied extracts affirmed the presence of evernic acid, physodalic acid, and protocetraric acid as major phenolic compounds. The biological activities of evernic and protocetraric acids have been reported in previous studies, where they showed higher activity than the one obtained for our examined lichen extracts including the antioxidant effect by DPPH assay. Also, they were found to exhibit strong antimicrobial activity against different microorganisms and anticancer activity against various cell lines [15, 29] while there are no available data on the antioxidant, antibacterial, and anticancer activities of physodalic acid [30].

In this study, the studied extracts demonstrated a relatively low cytotoxic activity against all cell lines used. This activity did not differ significantly from one line to another treated by the same extract, which means that these extracts reacted in the same way regardless of the cell used. These findings agree with the literature [14, 29, 30]. Among the extracts, *P. furfuracea* extract was found to induce the largest effect against all cancer cell lines tested, especially on human prostate cancer (22RV1) cell lines at exposure time 72 h. This result agrees with those reported in the previous study which showed that the extracts of *E. prunastri* and *P. furfuracea* possess cytotoxic effects against human melanoma (FemX) and human colon carcinoma (LS174) with similar IC_50_ values to ours (55.09–120.89 *µ*g/mL). Moreover, the strongest cytotoxic activity was presented by *P. furfuracea*. It was also determined that these extracts induced cell death caused by a strong arrest of the sub-G1 phase in the cell cycle of LS174 and FemX cells [15]. The literature also pointed out that either raw lichen extracts or their purified components were effective against different cancer cell lines even at low concentrations [16, 31, 32]. However, according to the American National Cancer Institute guidelines, the IC_50_ values of the three lichen extracts found in this study did not indicate strong cytotoxic activity IC_50_ > 30 *µ*g/mL [31], while the strong cytotoxic effect of physodic acid isolated from *P. furfuracea* vs. FemX and LS174 cancer cells with IC_50_ of 19.52 and 17.89 *µ*g/mL, respectively, was already reported [15].

Lichens have been involved in several studies looking for new natural antioxidants and their potential protective effects vs. chronic diseases [33, 34]. In the present work, our findings showed that the tested extracts had a potent *in vitro* antioxidant effect which correlated to content in total phenols. This result was in accordance with another published work which showed a positive correlation between the phenolic content and the antioxidant activity [14, 15]. Furthermore, no significant correlation between the flavonoid content in the lichen extract and the antioxidant effect was reported therein. This means that lichen components (depsides, depsidones, and dibenzofurans) are the principal agents responsible for the antioxidant activities. Among the tested extracts, *P. furfuracea* extract showed the best antioxidant power with the greatest concentration of polyphenolic compounds, which is in accordance with other studies carried out in acetone extract of *P. furfuracea* and *E. prunastri* harvested in Serbia and Turkey showing in the same way that *P. furfuracea* extract had a largest antioxidant activity and the highest quantity of phenols than *E.prunastri* extract [15, 35]. Our results indicated higher antioxidant capacity and phenolic content than those reported by Kosanić and Bìlgìn Sokmen in their studies. Moreover, our results showed that *R. farinacea* extract had the highest ferric reducing power, but the lowest phenols content, which suggests that this activity of this tested extract can be due to the presence of nonphenolic compounds.

Türka The antibacterial activity of *R. farinacea*, *P. furfuracea*, and *E. prunastri* extracts was evaluated by the microdilution method against bacterial strains including clinical isolates of methicillin-resistant *S. aureus*. The results relieved that all extracts exhibited a potent antibacterial effect vs. Gram-positive bacteria. However, no effect was observed for Gram-negative bacteria. These results are in harmony with those carried out by et al. [36] and Tay et al. [37] that reported a great activity of *P. furfuracea* and *R. farinacea* against only Gram-positive bacteria, and they also found that physodic acid and (+)-usnic acid isolated from these species, respectively, were inactive against Gram-negative strains [28, 29]. In recent a study, Gültekin and Özyiğitoğlu showed that acetone extract of *P. furfuracea* had no inhibitory effect on Gram-negative bacteria [38]; also, Osmana et al. reported that only Gram-positive bacteria were susceptible to the acetone extract of *P. furfuracea* and *Evernia divaricata* [39]. In contrast, other studies showed that these species have presented antibacterial effects vs. both Gram-positive and Gram-negative bacteria with stronger inhibitory effects on Gram-positive bacteria [22, 32]. The reason for these conflicting results may be due to variations in the genotype of the strains tested and the experimental conditions.

The high sensibility of Gram-positive bacteria might be interpreted by the fact that the structures of the cell envelope are different between both Gram-positive and Gram-negative bacteria. The former has an outer membrane formed by an inner phospholipid layer surmounted by LPS (lipopolysaccharide) macromolecules which prevent the diffusion of hydrophobic compounds. Without an outer membrane, the cell wall of Gram-positive bacteria can be easily permeable [40].

Finally, our research findings provided that the lichen extract tested demonstrated high antibacterial activity against MRSA clinical isolates from burn wounds. The acetone extracts of *P. furfuracea* and *E. prunastri* exhibited high activity with MICs ranged from 0.039 to 0.15 mg/mL and a bacteriostatic effect. Furthermore, *R. farinacea* extract exhibited a bactericidal effect against one MRSA with MIC values ranging from 0.078 to 0.625 mg/mL for all MRSA strains. This activity could be induced by usnic acid which was the major antibacterial agent in *R. farinacea* [37]. Pompilio et al. demonstrated that usnic acid showed significantly higher activity against MRSA strains than atranorin and fumarprotocetraric acid [41]. Other data indicated that usnic acid presented high antibacterial activity against clinical isolates of MRSA with MIC values ranging between 25 and 50 *μ*g/mL by disruption of the bacterial membrane [42]. Various lichenic compounds such as lobar acid, physodic acid, rhizocarpic acid, 3-hydroxyphysodic acid, hybocarpone, and (R)-(+)-usnic acid isolated, respectively, from *Sterocaulon dactylophyllum, Hypogymnia physodes, Psilolechia lucida, Hypogymnia physodes, Lecanora conizaeoides,* and *Lecanora albescens* lichen species were found to be effective vs. methicillin- and multidrug-resistant *Staphylococcus aureus* [43]. Despite, the antibacterial activity of lichens, either as raw extracts or purified compounds, was widely investigated, and the mechanism of action of these substances has not been sufficiently assessed [44].

## 5. Conclusions

The current study sheds light on the biological properties of extracts from *R. farinacea*, *E. prunastri*, and *P. furfuracea* growing in Morocco. The results reported here pointed out that the three lichen extracts possess significant antioxidant and antibacterial activities. *Pseudevernia furfuracea* extract exhibited the best antioxidant power, as well as the highest total phenolic content. The results also demonstrated that all studied extracts have antibacterial effects against only Gram-positive bacteria, especially against MRSA strains, with the highest activity was presented by the extract of *Pseudevernia furfuracea*. Therefore, the Moroccan lichens could be a promising source of bioactive natural products with a pharmaceutical interest. However, complementary studies should be conducted to identify the major metabolites that are responsible for this biological activity and their mechanism of action.

## Figures and Tables

**Table 1:** IC50 values of growth inhibitory effects of *R. farinacea*, E*. prunastri*, and *P. furfuracea* versus CHO, HT-29, Hep-G2, and 22RV1 cell lines at exposure time 72 h.

Lichen species	Growth inhibitory effects (IC50 (*µ*g/mL))			
	CHO	HT-29	Hep-G2	22RV1
*P. furfuracea*	63.60 ± 2.98^a^	57.10 ± 2.83^a^	68.60 ± 3.77^a^	42.30 ± 2.55^a^
*E. prunastri*	60.80 ± 0.36^a^	105.52 ± 0.79^b^	95.71 ± 1.50^ab^	103.80 ± 18.40^a^
*R. farinacea*	140.24 ± 10.40^b^	127.677 ± 5.835^c^	110.15 ± 18.50^b^	96.42 ± 16.40^a^
Mytomicine	3.80 ± 0.10^c^	0.90 ± 0.10^d^	3.21 ± 2.40^c^	2.56 ± 0.10^b^

Data are expressed in means (*n* = 3) ± SD. Values reported in the same column with different letters (a–d) significantly differ at *p* < 0.05.

**Table 2:** Total phenolic and flavonoids contents in *R. farinacea*, *E. prunastri*, and *P. furfuracea* extracts.

Lichen species	TPC (*μ*g GAE/mg of dry extract)	TFC (*μ*g CE/mg of dry extract)
*R. farinacea*	167.67 ± 50.20	17.63 ± 1.11^∗^
*E. prunastri*	194.33 ± 7.50	13.50 ± 2.14
*P.furfuracea*	328.67 ± 26.81^∗∗^	12.23 ± 0.40

Data are reported as mean (*n* = 3) ± SD, ^∗^: *p* < 0.05, ^∗∗^:*p* < 0.01.

**Table 3:** IC50 values of crude extracts of *R. farinacea*, *E. prunastri*, and *P. furfuracea*.

Species	IC50 (*µ*g/mL)
*R. farinacea*	>1000
*E. prunastri*	>1000
*P. furfuracea*	498.40 ± 44.14
Ascorbic acid	8.90 ± 0.10^∗∗^
Trolox	17.83 ± 0.50^∗^

Data are reported as mean (*n* = 3) ± SD, ^∗^: *p* < 0.05, ^∗∗^:*p* < 0.01.

**Table 4:** *In vitro* antibacterial effect of crude extracts of *P. furfuracea*, *E. prunastri*, and *R. farinacea*.

Bacteria	*Pseudeverina furfuracea*			*Evernia prunastri*			*Ramalina farinacea*		
	MIC^a^	MBC^a^	MBC/MIC	MIC^a^	MBC^a^	MBC/MIC	MIC^a^	MBC^a^	MIC/MBC
*Bacillus subtilis*	0.078	0.625	8.012	0.078	1.25	16.025	0.078	0.625	8.012
*Listeria innocua*	0.31	0.625	2.016	0.625	2.5	4	0.31	1.25	4.032
*Staphylococcus aureus*	0.078	0.625	8.012	0.078	0.625	8.012	0.15	1.25	8.333
MRSA N°1	0.039	0.625	16.02	0.039	0.625	16.02	0.15	0.625	4.16
MRSA N°2	0.15	0.625	4.16	0.15	1.25	8.33	0.625	1.25	2
MRSA N°3	0.078	0.625	8.01	0.15	1.25	8.33	0.625	1.25	2
MRSA N°4	0.15	0.625	4.16	0.15	0.625	4.16	0.625	1.25	2
MRSA N°5	0.078	0.625	8.012	0.15	0.625	4.16	0.625	1.25	2
*Escherichia coli*	>25	—	—	>25	—	—	>25	—	—
*Pseudomonas aeruginosa*	>25	—	—	>25	—	—	>25	—	—
*Proteus mirabilis*	>25	—	—	>25	—	—	>25	—	—

a: (mg/mL), MRSA: Methicillin-Resistant *Staphylococcus aureus*.

## Data Availability

All data are incorporated in the manuscript.
